# Collagen-based silver nanoparticles for biological applications: synthesis and characterization

**DOI:** 10.1186/s12951-014-0036-6

**Published:** 2014-09-17

**Authors:** Vinicius S Cardoso, Patrick V Quelemes, Adriany Amorin, Fernando Lucas Primo, Graciely Gomides Gobo, Antonio C Tedesco, Ana C Mafud, Yvonne P Mascarenhas, José Raimundo Corrêa, Selma AS Kuckelhaus, Carla Eiras, José Roberto SA Leite, Durcilene Silva, José Ribeiro dos Santos Júnior

**Affiliations:** Research Center in Biodiversity and Biotechnology (Biotec), Campus Parnaíba, Federal University of Piauí, Av São Sebastian 2819, 64202-020 Parnaíba, Piauí Brazil; Physiotherapy Department, Campus Parnaíba, Federal University of Piauí, Av. São Sebastião 2819, 64202-020 Parnaíba, Piauí Brazil; Departamento de Química, Laboratório de Fotobiologia e Fotomedicina, Faculdade de Filosofia, Ciências e Letras de Ribeirão Preto, Universidade de São Paulo, 14040-901, Ribeirão Preto, SP Brazil; Institute of Physics of São Carlos (IFSC), University of São Paulo (USP), 13566-590 São Carlos, SP Brazil; Laboratory of Microscopy, Institute of Biology, University of Brasília, 70910900 Brasília, DF Brazil; Area of Morphology, Faculty of Medicine, University of Brasília, Brasília, 70910900 DF Brazil; Interdisciplinary Laboratory for Advanced Materials (LIMAV), Federal University of Piauí, 64049-550 Teresina, PI Brazil; Department of Chemistry, Campus Teresina, Federal University of Piauí, 64049-550 Teresina, Piauí Brazil

**Keywords:** Silver nanoparticles, Collagen, Antimicrobial activity, Cell viability

## Abstract

**Background:**

Type I collagen is an abundant natural polymer with several applications in medicine as matrix to regenerate tissues. Silver nanoparticles is an important nanotechnology material with many utilities in some areas such as medicine, biology and chemistry. The present study focused on the synthesis of silver nanoparticles (AgNPs) stabilized with type I collagen (AgNPcol) to build a nanomaterial with biological utility. Three formulations of AgNPcol were physicochemical characterized, antibacterial activity *in vitro* and cell viability assays were analyzed. AgNPcol was characterized by means of the following: ultraviolet–visible spectroscopy, dynamic light scattering analysis, Fourier transform infrared spectroscopy, atomic absorption analysis, transmission electron microscopy and of X-ray diffraction analysis.

**Results:**

All AgNPcol showed spherical and positive zeta potential. The AgNPcol at a molar ratio of 1:6 showed better characteristics, smaller hydrodynamic diameter (64.34 ± 16.05) and polydispersity index (0.40 ± 0.05), and higher absorbance and silver reduction efficiency (0.645 mM), when compared with the particles prepared in other mixing ratios. Furthermore, these particles showed antimicrobial activity against both *Staphylococcus aureus* and *Escherichia coli* and no toxicity to the cells at the examined concentrations.

**Conclusions:**

The resulted particles exhibited favorable characteristics, including the spherical shape, diameter between 64.34 nm and 81.76 nm, positive zeta potential, antibacterial activity, and non-toxicity to the tested cells (OSCC).

**Electronic supplementary material:**

The online version of this article (doi:10.1186/s12951-014-0036-6) contains supplementary material, which is available to authorized users.

## Background

Collagen is the most abundant protein constituting to the 30% of total protein and 6% of animal body weight [1,2]. Type I collagen, a natural polymer, is a major extracellular matrix protein in mammals and exhibits favorable characteristics for promoting cell proliferation [3-5]. It can influence the cell physiology and morphology [4,6], create a good matrix for endothelial cells *in vitro,* induce platelet aggregation, promote blood clotting, and consequently accelerate the healing of skin wounds [7].

Since 1980s, some scientists have been using collagen as a matrix to regenerate tissues for repairing skin [8], bone [9], knee meniscal [10], joint cartilage [11], esophagus [12], dura mater [13], muscle [14] and nervous system [15]. The use of collagen combined with glycosaminoglycans as a skin implant has been already tested [16,17]. The ability of collagen gel to regenerate cornea and nerves has been also demonstrated by recent animal studies and clinical trials [18,19]. Furthermore, it has been shown that the combined collagen and hyaluronic acid can promote the revascularization of tissues in animal models [20].

In the field of nanotechnology, collagen scaffold has been widely used in biological experiments for introducing chemical and pharmaceutical substances. Bakare et al. [21] proposed a method for constructing a film by using poly(hydroxybutyrate valerate) (PHBV) grafted with scaffold tipo I collagen to support silver nanoparticles (AgNPs). Jithendra et al. [22] suggested a blend of *Aloe Vera* with collagen and chitosan scaffold for tissue engineering applications.

Metal nanoparticle, especially those made of noble metals, show excellent properties for biotechnology applications [23–25]. In particular, AgNPs have established a broad range of applications in the majority of biomedical studies [26], due to their antibacterial ability and selective toxicity to microorganisms [27].

In addition, AgNPs are widely used in various medical and industrial fields for venous catheters coating; vascular prostheses manufacturing; wound dressing manufacturing; treatment for chronic wounds and ulcers [25]; or as a constituent incorporated into cement for the realignment of bone fractures [27], in to water purification filter [28] and into wall paint for providing an aseptic environment to hospital patients [29].

The ability of AgNPs to control bacterial activity relies on the interactions with three major structural components of the bacteria: namely peptidoglycan in the cell wall, DNA, and proteins, by mainly affecting the enzymes involved in the electron transport chain [30–33].

The ideal properties of AgNPs for biomedical applications include prolonged effectiveness, high levels of bactericidal and bacteriostatic activity, ability to prevent a broad spectrum of bacteria, high biocompatibility, and low toxicity *in vivo* [33]. In particular, the shape and concentration of AgNPs in solutions are important factors in ensuring the effective contact of the particles with the bacterial membranes and in determining the amount of AgNPs for effectively inhibiting the targeting bacteria [34].

Some literatures reported the application of AgNPs for treating the wounds of mice, and these particles showed excellent tensile properties and resulted in improved alignment of fibers for skin repair [35,36].

Based on the previously discussed properties and applications of collagen and AgNPs, we designed and synthesized three types of AgNPs stabilized with type I collagen (AgNPcols) by using a chemical synthesis route in the present study. This article presents their chemical synthesis, physicochemical characterization, analysis of activity against gram-positive and gram-negative bacteria, and *in vitro* cell viability assays.

## Results and discussion

Type I collagen is the most abundant protein in mammals and is present during tissue repair [1–5,7]. Although collagen has been used in biomedical research for several years, AgNPs stabilized with collagen, as well as their biocompatibility and antibacterial properties, have been recently reported by Alarcon et al. [37]. The authors used a photochemical route for fabricating AgNPs from silver nitrate (AgNO_3_), and this route was different from the chemical route employed in this study, where a reducing agent, sodium borohydride (NaBH_4_), was involved. Because NaBH_4_ is unstable when being in contact with water at room temperature, it is necessary to stabilize NaBH_4_ by using ultra-pure water at low temperature (4°C) and keep the solution refrigerated until use. In addition, Sun et al. [38] reported the use of NaBH_4_ for the synthesis AgNPs associated to a trisodium citrate solution. Thereafter, a multilayer film consisting of AgNPs and collagen in a layer-by-layer (LbL) configuration is generally constructed for stabilizing the particles.

An exclusive study on AgNPs stabilized by collagen has been reported [37]. Based on this study, we designed and synthesized three different formulations of AgNPs, at AgNO_3_ to NaBH_4_ molar ratios of 1:1, 1:6, and 1:15, by varying the concentration of NaBH_4_ to obtain the best silver (Ag^0^) reduction result in solution. The solution at the AgNO_3_/NaBH_4_ molar ratio of 1:6 resulted in a final Ag^0^ concentration of 0.64 mM (Table 1), as confirmed by the atomic absorption test. In the ratio of 1:1 between AgNO_3_ and NaBH_4_, the amount of reducer was not sufficient to reduce all molecules of silver. At ratio of 1:6 was obtained the best concentration for the chemical reaction, probably the molecules amount of AgNO_3_ and NaBH_4_ reached an optimum value for reduction. However, the ratio was 1:15 excess NaBH_4_ causing release of ions in solution and forming nanoparticles with hydrodynamic diameter higher by aggregation [39].

**Table 1:** **Diameter, zeta potential, PDI of AgNPcols (mean ± standard deviation) and molar concentration of silver in the solution**

	**Diameter (nm)**	**Zeta Potential (mv)**	PDI*	**[Ag] (mM)**
AgNPcol (1:1)	78.87 ± 12.89	31.8 ± 0.62	0.60 ± 0.02	0.434
AgNPcol (1:6)	64.34 ± 16.05	24.9 ± 0.79	0.10 ± 0.05	0.645
AgNPcol (1:15)	81.76 ± 18.22	19.9 ± 0.4	0.77 ± 0.17	0.345

*PDI: polydisperity index.

**Figure 1:**
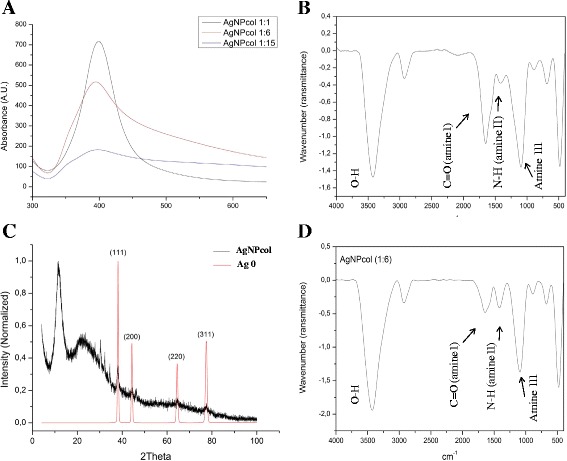
**AgNPcol characterization. (A)** Absorbance spectra of AgNPcols at three different NaBH_4_ to AgNO_3_ molar ratios; **(B)** FTIR spectra of collagen; **(C)** XDR patterns of AgNPcol (1:6 molar ratio); **(D)** FTIR spectra of AgNPcol (1:6 molar ratio).

All synthesized solutions were characterized in terms of particle size, zeta potential, and polydispersity index (PDI) by dynamic light scattering (DLS) analysis. A positive potential (19.9–31.8 mV) was obtained for all AgNPcols (Table 1). This occurs due to amino group carries a positive charge and is present in AgNPcol [40,41] (Figure 1D). The hydrodynamic diameter of the nanoparticles was between 64.34 nm and 81.76 nm and PDI value was between 0.40 and 0.77. The AgNPs associated with titanium dioxide synthesized by Desai and Kowshik [42] showed PDI value of 0.47, which was very close to the result obtained from this study, although other PDI values were also reported [43–45]. The value of PDI is one of the major parameters used for selecting a low-polydispersity solution for the subsequent cell viability test.

The presence of a positive zeta potential favors the interaction between the particles and Gram-negative and Gram-positive bacteria [46]. The efficiency of ionic silver against bacteria with negatively charged membranes is related to the electrostatic attraction caused by the positive potentials of the particles [47]. In the present study, the positive zeta potential of AgNPcols is one of the aspects that may explain the favorable result of its acting against *E. coli* and *S. aureus*. Hamouda and Baker [48] reported that the opposite surface charges could promote the interactions between the bacterial membranes and AgNPs. They also mentioned that, due to the small size of nanoparticles, the tested solutions could easily permeate the membranes of the bacteria and promote their death [34,49]. Furthermore, Baker et al. [50] reported that small particles with large contact areas showed increased efficiency against bacteria, as compared with the particles with large sizes. Saptarshi et al. [51] suggests that associate protein with nanoparticle, there are better cell absorption because the protein favors interaction with cell membrane facilitating interaction to nanoparticle with bacteria and another live cells.

The increase in the proportion of NaBH_4_ during particle synthesis could reduce the zeta potential of the resulting particles. Zhang and Wu [39] reported the same behavior of gold nanoparticles and claimed that this was due to the aggregation of metal particles. The AgNPcols produced in our study demonstrated similar behaviors, as indicated by the decrease in the amount of nanoparticles in the solution. This is because that the aggregated Ag^0^ formed larger particles, resulting in a polydisperse solution and precipitation during centrifugation. The relationship between the increases of the ratio of NaBH_4_ in relation to the increase in diameter of the nanoparticles is due to the release of electrons caused by NaBH_4_. Because when an increase occurs in the concentration of NaBH_4_ increases the number of free electrons in the solution and decreases the zeta potential favors the aggregation of silver [39].

**Figure 2:**
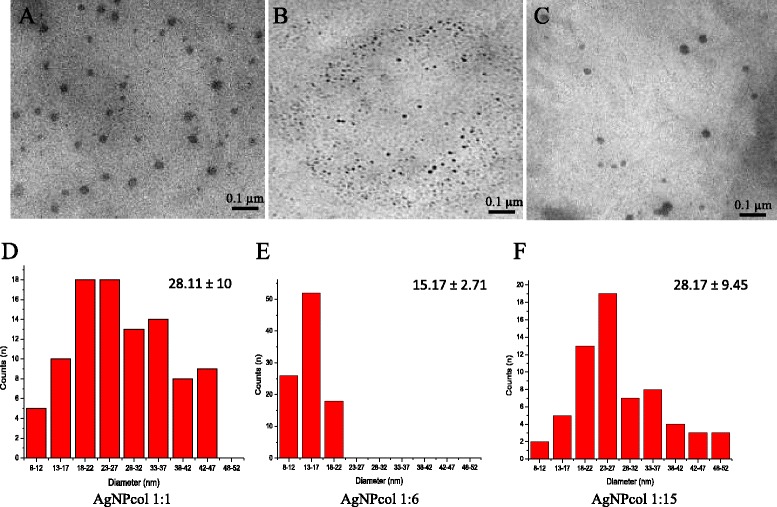
**Images of Silver nanoparticle stabilized with collagen.** TEM images of AgNPcol at AgNO_3_/NaBH_4_ molar ratio of **(A)** 1:1, **(B)** 1:6, and **(C)** 1:15 molar ratio. Histograms showing the particle size distribution of AgNPcol at molar ratio of **(D)** 1:1 (28.11 ± 10 nm), **(E)** 1:6 (15.17 ± 2.71 nm), and **(F)** 1:15 (28.17 ± 9.45 nm) (scale bar = 0.1 μm).

After synthesis, all solutions were characterized by using ultraviolet–visible (UV–vis) spectroscopy analysis, which was efficient for detecting the sensitive AgNPs that could display a strong absorption peak [52,53]. In this study, we found a wider plasmon band and lower absorption peak intensity for the solution at a higher NaBH_4_ to AgNO_3_ molar ratio, as compared with the solutions at lower molar ratios (Figure 1A). It is believed that this is due to the aggregation of Ag^0^ molecules [53,54], as indicated by the values of zeta potential described above and the presence of large particles in AgNPcol at molar ratio of 1:15. We can also notice the presence of the plasmon band between 380 nm and 450 nm, and this is indicative of the spherical shape of AgNPs [55], which can be further confirmed by transmission electron microscopy (TEM) analysis (Figure 2). Some researchers [35,56] reported that the spherical shape is the optimal morphology for nanoparticles against bacteria, as it can facilitate the interaction between the particles and the bacterial membranes.

The difference in size AgNPcol found between the results of Table 1 (DLS) and Figure 2 (MET) occurs because the DLS diameter is measured in solution (hydrodynamic diameter value). Already in the TEM, the nanoparticle is no in solution and the result is a projected estimate of the diameter of the nanoparticle. The hydrodynamic diameter of the nanoparticles (DLS) was highlight, because this nanoparticle was developed for use in a biological environment and will be in solution [57].

From the Fourier transform infrared spectra (FTIR) of collagen and AgNPcol (1:6 molar ratio) (Figure 1B and D), we can observe the presence of C = O (amine I at 1652.84 cm^−1^) and NH bands (amine II at 1571.64 cm^−1^) in both samples of collagen and AgNPcol. However, the band due to C = O (amine I at 1652.84 cm^−1^) in the spectrum of AgNPcol showed a low intensity, indicating that this group was possibly involved in the reduction and stabilization of AgNPs. Sun et al. [38] suggested that these changes were due to the association of collagen molecules (i.e., amines) with AgNPs.

The phases of the samples were determined by X-ray powder diffraction (XDR) analysis by searching against databases. In Figure 1C, it can see the silver peaks (ICSD: 44387-Ag0), with reflections identified. Traces of silver oxides (ICSD: 35540-Ag2O, 27659-AgO, 15999/59193-Ag2O3, 202218-Ag3O4) were also identified from the sample. Silver oxides reflections can be found in [Additional file 1]. This result is consistent with our expectation that oxidation products can be formed on the surface of pure silver. In addition, we found that the amorphous phase of collagen affected the sample’s crystallinity, resulting in its semi-crystalline state.

The activity of AgNPcols against the gram-positive bacterium, *Staphylococcus aureus* (ATCC 29213), and gram-negative bacterium, *E. coli* (ATCC 25922), was tested by using an AgNO_3_ solution as control. Based on the results of the atomic absorption spectrometric study, the concentration of AgNPcols was corrected for the antimicrobial assays. It was found that the behavior of AgNPcol (1:6 molar ratio) for inhibiting the growth of bacteria was comparable to that of AgNO_3_ (Table 2). Thus, we may assume that the silver particle can maintain its antimicrobial property when being incorporated into the collagen-stabilized nanoparticles. In Alarcon’s study [37] about AgNPs stabilized with collagen, was lower than that of AgNO_3_ used as control.

**Table 2:** **Minimum inhibitory concentrations (MICs) of AgNPcol (μg Ag/mL), AgNO**_**3**_ **(μg Ag/mL), and standard antibiotics (μg/mL) for inhibiting** ***Staphylococcus aureus*** **and** ***Escherichia coli***

	**AgNPcol (μgAg/mL)**			**Controls**	
Bacterial strains	1:1	1:6	1:15	AgNO_3 (μgAg/mL)_	Antibiotic _(μg/mL)_
*S. aureus*	11.7	17.4	11.7	13.5	<0.5^a^
*E. coli*	11.7	8.7	11.7	6.75	<0.5^b^

^a^Oxacilin.

^b^Meropenen.

**Figure 3:**
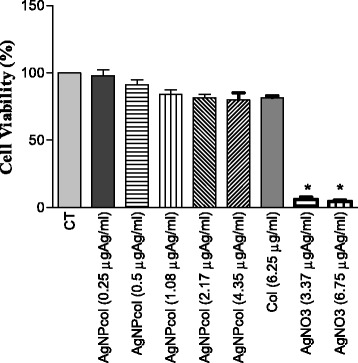
**Cell viability.** Results of cell viability test of AgNPcol and control solutions (Col and AgNO_3_). All data were expressed as mean ± SEM values of three independent experiments. A value of *p < 0.05 was considered statistically significance.

For the cell viability test, we chose only one synthesized solution by analyzing the relevant characterization data. AgNPcol at the molar ratio of 1:6 was chosen for the test, due to its smaller particle size, lower PDI, and a higher percentage of Ag^0^ than those of other samples, as determined by atomic absorption spectroscopy. The results (Figure 3) indicated that the AgNPcol solution at the tested concentrations did no cause significant differences in cell viability as compared with the control (CT). In addition, AgNO_3_ and collagen (Col) were also used for comparison. It is known that collagen does not show any cytotoxicity towards cells, as this can be evidenced by its abundance in animals and the human body. Although AgNO_3_ solution was reported to be toxic to cells, the AgNPcol at the approximate Ag concentration as that of AgNO_3_ did not show any toxicity to the cells tested in this study. We attempted to use higher concentrations of AgNPcol to evaluate its cytotoxicity; however, precipitation occurred in the solution before incubation under the physiological pH and ambient temperature conditions.

Gurunathan et al. [58] performed the cell viability test by using breast cancer cells (MDA-MB-23) and AgNP at 5 μg/ml, which did not exhibit cytotoxicity as compared with the control. In addition, Prokopovich et al. [59] synthesized the AgNPs by using NaBH_4_, and the produced nanoparticles were incorporated into the bone cement, which did not show cytotoxicity to osteoblast cells (MC 3TC) either.

The results of the present study showed that the synthesized AgNPcol was effective against the tested bacteria and was non-toxic to the examined cells. Further tests will be conducted to evaluate the *in vivo* cytotoxicity and healing ability of AgNPcol by using biological tissues and animal samples.

## Conclusion

In the present study, we demonstrated the synthesis of an AgNP solution stabilized with type I collagen by using NaBH_4_ as a reducing agent. The resulted particles exhibited favorable characteristics, including the spherical shape, diameter between 64.34 nm and 81.76 nm, positive zeta potential, antibacterial activity, and non-toxicity to the tested cells (OSCC). It is found that the activity against bacterium is facilitated by the electrostatic interaction between the positively charged AgNPcols and the negatively charged bacterial membranes. Probably the shape, size and positive zeta potential of AgNPcols facilitates the activity against gram negative bacterium and gram positive bacterium. Furthermore, the cell viability test provides the basics for the future study that aims to investigate the *in vivo* behaviors of AgNPcols by using biological tissues.

## Methods

### Synthesis of collagen-based silver nanoparticles (AgNPcols)

A solution of silver nitrate (AgNO_3_) at a concentration of 108 μgAg/mL, a collagen type I from rat tail (Santa Cruz Biotechnology) solution at a concentration of 0.1 mg/ml and a solution of borohydride (NaBH_4_) at 3.78 mg/ml, prepared using ultrapure water at 4°C were used to carry out the synthesis of nanoparticles.

The AgNO_3_ solution was added to the collagen, both with the same volume and remained under agitation to homogenize for 10 min. The NaBH_4_ solution was added later, in the form of jet, for any solution of NaBH_4_, came in contact with the Beker solutions quickly and completely. This solution was stirred for 10 minutes to homogenize. Subsequently, the reaction mixture was centrifuged at 3600 rpm for 15 minutes and finally separated from the supernatants of the final solution present in the container. In the present study three different proportions of borohydride solution regarding Silver Nitrate (AgNO_3_) (molar ratio: 1:1, 1:6 e 1:15) were selected.

### Physicochemical characterization of AgNPcols

The AgNPcols were characterized by UV–vis spectroscopy using a Shimadzu (UV 1800) spectrophotometer. Were subsequently characterized according to their size, electrical potential and PDI using the DLS (Malvern Zetasizer Nano ZS Model 3600) with laser with a wavelength of 633 nm and scattering angle of 90° all measurements were performed in triplicate. To verify the shape and confirm the diameter of the nanoparticles non-diluted samples were placed on two screens (20 μL) for transmission electron microscopy (TEM) previously coated with Formvar. After drying for 2 h at room temperature (25 ± 2°C) screens were analyzed in a Jeol JEM-1010 electron microscope and photomicrographed by an UltraScan® with Digital Micrograph 3.6.5 software (Gatan/USA) [25].

In order to quantify the percentage of silver in solution, the atomic absorption spectroscopy (Varian - Model AA240FS) was used, with a wavelength of 328.1 nm and multielement lamp (Varian No. 5610108700). The reading was held in atomic absorption flame with Oxygen and Acetylene gases.

XRD data were obtained at the Laboratory of X-ray Crystallography of IFSC/USP using a Rigaku Rotaflex diffractometer equipped with graphite monochromator and rotating anode tube, operating with Cu Ka, 50 kV and 100 mA. Powder diffraction patterns were obtained in step scanning mode, 2θ = 5–100°, step of 0.02° and 5 s/step. Peak Fitting Module program [60] was used for the peak decomposition of the semicrystalline pattern and determination of area due to the amorphous phase.

### Evaluation of antibacterial activity of AgNPcols

To study the antibacterial properties of *AgNPcols*, by the determination of Minimum Inhibitory Concentration (MIC), two bacterial strains were selected: *Staphylococcus aureus* ATCC 29213 (Gram-positive) and *Escherichia coli* ATCC 25922 (Gram-negative). The microorganisms were cultured in Mueller-Hinton agar at 37°C for 24 hours in aerobic conditions. Then a suspension of bacterial strains with an optical density of McFarland of 0.5 (1 × 10^8^ CFU/mL) was made in an isotonic sodium chloride 0.85% solution. Later in time, this solution was diluted ten times (1 × 10^7^ CFU/mL) and used as inoculum in the experiment. MIC was determined according to protocols previously described [61–63] using 96-well microdilution plate with Mueller-Hinton broth where the strains (concentration of 5 × 10^5^ CFU/mL) were exposed to two-fold dilution series of the AgNPcols with concentrations ranging from 34,8 to 0,36 μgAg/mL. The same procedure was used to determine the MIC of the following controls: collagen, AgNO_3_, and standard antibiotics effective against the tested bacterial strains with concentrations ranging from 27 to 0.42 μgAg/mL for AgNO_3_; 50 to 3,12 μg/mL for collagen and 32 to 0.5 μg/mL for antibiotics. Sterile Mueller-Hinton broth was used as the negative control and inoculated broth was used as the positive control. MIC was defined as the lowest concentration of agent that restricted the visual bacterial growth in the culture media.

### Cell viability

For the study of cell viability, the AgNPcol 1:6 was diluted four times with the ratio of two, starting at a concentration of 4.35 μgAg/ml. The cell lines used in this study was oral squamous cell carcinoma (OSCC) obtained from American Type Culture Collection (ATCC, Manassas, VA). Cells were grown in 75 cm^2^ flasks and maintained in Dulbecco’s modified Eagle medium (DMEM) supplemented with 10% fetal bovine serum, streptomycin/penicillin antibiotics and non-essential aminoacids. For the experiment the cells were seeded in a 96-well plate (5,000 cel./Well) and kept in an incubator (atmosphere at 37°C and humidified 5% CO_2_) for 24 hours. In a 96-well plate was added 5 different concentrations AgNPcol solutions and control solutions (medium, collagen - 6.25 μg/ml, AgNO3 - 3.37 μgAg/ml and AgNO3 - 6.75 μgAg/ml). The plates were kept in an incubator for 3 hours. Subsequently, the added solutions were removed and placed in Hank's buffer for 5 minutes. The buffer was removed and fresh medium was added. After 24 hours the medium was removed and added to a solution of DMEM without phenol and MTT ([3-(4,5-dimethyl thiazol-2-yl)-2,5-*diphenyl tetrazolium bromide*) (15%) to each well. The plates were incubated for 4 hours. Thereafter all the medium with MTT was removed with care to do not remove formazam produced by living cells. Finally, isopropanol was added to each well to solubilize the formazam and taken to review the reader Safire (TECAN EUA Inc., Durham, NC). Statistical analysis was performed using Prism 5.0 (GraphPad Software) by ANOVA and Tukey test. All data were expressed as mean and standard deviation of three independent experiments. We used the statistical significance of p <0.05 for this study [64].

## Additional file

Additional file 1
**Additional information about XRD result.**

## Abbreviations

AgNPs: Silver nanoparticles
AgNPcols: Silver nanoparticles stabilized with type I collagen
AgNO_3_: Silver nitrate
NaBH_4_: Sodium borohydride
LbL: Layer-by-layer
Ag^0^: Silver
mM: Millimolar
PDI: Polydispersity index
UV–vis: Ultraviolet–visible
DLS: Dynamic light scattering
TEM: Transmission electron microscopy
FTIR: Fourier transform infrared spectra
XDR: X-ray powder diffraction
MICs: Minimum inhibitory concentrations
CT: Control
Col: Collagen
